# Impact of endoscopic thoracic R4 sympathicotomy combined with R3 ramicotomy for primary palmar hyperhidrosis

**DOI:** 10.3389/fsurg.2023.1144299

**Published:** 2023-02-24

**Authors:** Yunhe Huang, Yunkun Liu, Wei Zou, Na Mao, Jian Tang, Lei Jiang, Guowen Zou, Lun Yang, Bentong Yu, Guangxia Wei

**Affiliations:** Department of Thoracic Surgery, the First Affiliated Hospital of Nanchang University, Nanchang, China

**Keywords:** primary palmar hyperhidrosis, endoscopic thoracoscopic sympathectomy, ramicotomy, compensatory hyperhidrosis, rami communicantes

## Abstract

**Background:**

Endoscopic thoracoscopic sympathectomy (ETS) is the preferred method for treating primary palmar hyperhidrosis (PPH) that bears the risk of compensatory hyperhidrosis (CH) following surgery. The current study aims to evaluate the effectiveness and safety of an innovative surgical procedure of ETS.

**Methods:**

A survey of the clinical data of 109 patients with PPH who underwent ETS in our department from May 2018 to August 2021 was retrospectively conducted. The patients were organized into two groups. Group A underwent R4 sympathicotomy combined with R3 ramicotomy. Group B underwent R3 sympathicotomy. Patients were followed up to evaluate the safety, effectiveness and the incidence of postoperative CH of the modified surgical approach.

**Results:**

A total of 102 patients completed follow-up, and seven of the total enrolled patients were lost to follow-up, with a loss rate of 6% (7/109). Among these, Group A constitutes 54 cases, group B constitutes 48 cases, and the mean follow-up was 14 months (interquartile range 12–23 months). There was no statistically difference in surgical safety, postoperative efficacy, and postoperative quality of life (QoL) score between group A and group B (*p* > 0.05). The score of the psychological assessment was higher (*p* = 0.004) in group A (14.15 ± 2.06) compared to group B (13.30 ± 1.86). The incidence of CH in group A was lower than in group B (*p* = 0.019).

**Conclusion:**

R4 sympathicotomy combined with R3 ramicotomy is safe and effective for PPH treatment, along with a reduced incidence of postoperative CH rate and improved postoperative psychological satisfaction.

## Introduction

PPH, one of hyperhidrosis, is a non-organic benign disease characterized by excessive sweating on the local palm, which may also be accompanied by hyperhidrosis of other locations including the craniofacial region, axilla, chest, abdomen, back, and planta (1). The incidence of PPH is 2.1% in China (2), being particularly prevalent among adolescents. Lai proposed three levels of palmar hyperhidrosis, according to the manifestations of sweaty palms, mild (palms were slightly wet), medium (sweaty palms that drench a handkerchief), severe (sweaty palms that may cause autonomic dripping) (2). Although PPH is rarely fatal, severe cases can have a significant impact on a person's ability to learn, work, and live a quality life. Furthermore, severe PPH will also interrupt patients' normal social activities, leading to social introversion, avoidance, fear and so on. And may eventually result in severe depression, anxiety, and other psychological disorders in the long run.

The treatment for PPH comprises surgical and conservative traditional clinical therapies. Conservative clinical therapies contain skin application of antiperspirant agents, anticholinergic drugs, iontophoresis, Botulinum injections, photodynamic therapy and others (3–7). Regardless, all of these therapies have a temporary duration of effect, and some drugs may generate serious side effects if used for an extended period of time. ETS is widely accepted by the vast majority of patients for the benefits of good efficacy and high safety. It is the common first choice for PPH surgical treatment, as suggested by the Society of Thoracic Surgeons (8).

CH is the most frequent postoperative complication of ETS. The question of how to safely acquire postoperative satisfaction while also efficiently controlling the occurrence of CH is still a concern understudying. This study estimates the safety and effectiveness of the modified surgical procedure for PPH and the efficacy of controlling CH occurrence by resembling it with traditional R3 sympathicotomy.

## Clinical data and methods

### Patients and methods

A retrospective research was conducted on patients with moderate to severe PPH who received ETS in the Department of Thoracic Surgery of the First Affiliated Hospital of Nanchang University from May 2018 to August 2021. This study included 109 patients divided into two groups. Group A with those who underwent bilateral thoracic R4 sympathicotomy combined with R3 ramicotomy and group B with those who received bilateral thoracic R3 sympathicotomy. Clinical data of 102 patients were gathered to estimate the improvement of palm sweating, complications, postoperative satisfaction, and quality of life (QoL) after excluding 7 lost ones. The study protocol was approved by our institutional review board (Approval No.: (2022)CDYFYYLK(06-020)).

### Surgical technique

**Figure 1:**
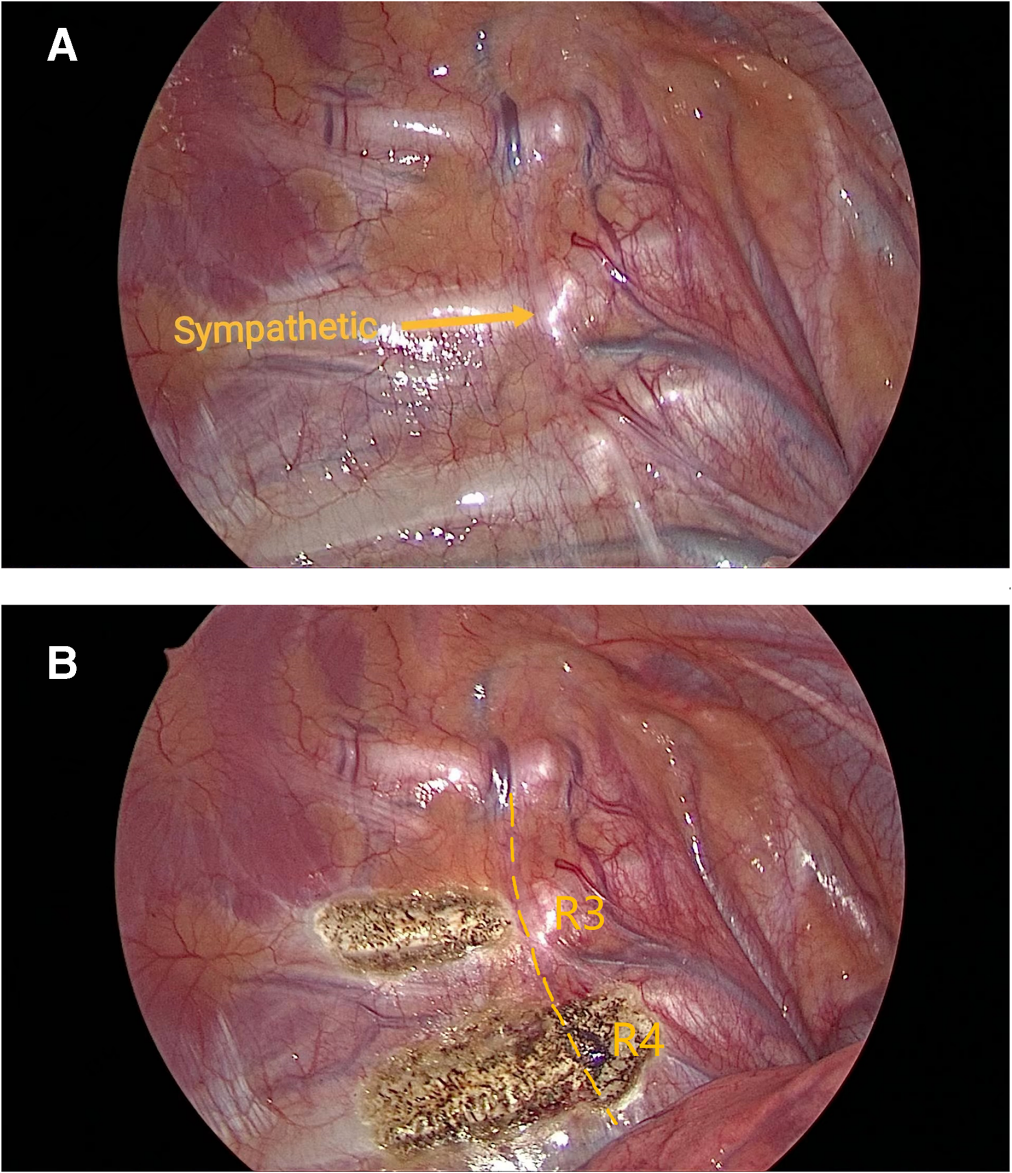
View under thoracoscope (**A**) pre-operation. (**B**) post-operation.

Under general anesthesia with single-lumen endotracheal tube, all patients were positioned in the semi-sitting supine position at 45° with both arms abducted to 90°. Trocar (5 mm) was placed in the 4th intercostal space on the bilateral anterior axillary line, a 5-mm camera was introduced and Trocar was removed. A microelectrocautery hook was inserted along the original incision upon the camera, and artificial pneumothorax and the respiratory suspension were used to keep pulmonary collapse. In group A, we sectioned the R3 rami communicantes by fulgurating 2-cm outward along the lateral edge of the sympathetic chain in 2–3 mm of the third rib, leaving R3 sympathetic chain untouched, then fulgurating R4 sympathetic chain and R4 rami communicantes along the fourth rib (Figure 1). In group B, R3 sympathetic chain and R3 rami communicantes were severed by fulgurating along the third rib. The incision was closed after lung aeration recruitment, and thoracic imaging tests were reexamined after surgery to ensure there was no evidence of a pneumothorax or hemothorax before patients were discharged from the hospital.

### Postoperative follow-up

Postoperative follow-up data were collected through regular visits in the outpatient reexamination and telephone surveys after surgery including surgical satisfaction, the incidence and severity of CH, and the palmar condition. This data was further classified into four grades: 1. Excessive dryness 2. Neither dry nor wet, normal palm sweating status, 3. Sweatier than normal palms, but substantially better than preoperative palms, 4. Recurrence: palm sweating is identical to or slightly improved to preoperative palms. The severity degree of CH was scaled from 1 to 4: (I) Mild, with moist skin, no excessive sweating and negligible discomfort, (II) Moderate, noticeable sweating and discomfort, but tolerable, (III) Severe, excessive sweating, which necessitated a change in clothes within a day, but tolerable, (IV) Extremely severe, excessive sweating, running sweat was greatly increased which affected the patient's QoL, with the feeling of unbearable and even regret about undergoing the operation. The QoL grading evaluated the postoperative quality of life of the patients. The quantitative score of the discomfort caused by CH was questioned with a total score of 10, the more intense discomfort with the higher score.

### Statistical analysis

For the statistical analysis, SPSS 25.0 (IBM Corp, NY, USA) statistical software was used. The continuous variables subject to normal distribution were presented in the form of Mean ± Standard deviation, differences were detected by Student's t-test and analyzed by variance (ANOVA), and statistical significance of categorical variables was assessed by *χ*^2^ test or Fisher's exact test. *P* < 0.05 was considered statistically significant.

## Results

### Clinical characteristics

The questionnaire was completed by 102 subjects in group A (54 cases) and group B (48 cases) out of a total of 109 eligible patients. The mean follow-up was 14 months (IQR 12–23 months). Out of these, two cases had a preoperative pacemaker implanted due to sinus bradycardia and a positive atropine test, showed no abnormalities during and after operation time, and safely removed the temporary pacemaker before discharged. One case experienced ventricular preexcitation and received ETS in our department three days after interventional radiofrequency ablation, observed no abnormalities in postoperative and post-discharge reviews. There were no significant differences between the two groups in terms of age, gender, BMI, complications, family history, and degree of PPH (*P* > 0.05) (Table 1). Among the 102 patients, 4 cases (3.9%) showed palmar hyperhidrosis, 44 cases (42.7%) had palmar combined plantar hyperhidrosis, 1 case (0.9%) had palmar combined axillary hyperhidrosis, 53 cases (53.7%) had palmar combined axillary and plantar hyperhidrosis, no cases accompanied with craniofacial hyperhidrosis as shown in Table 2.

**Table 1:** General characteristic of patients.

Characteristic	Group A	Group B	*P* value
Age (years old)	22.59 ± 6.14	22.67 ± 4.96	0.97
Gender			0.55
Male	30	27	
Female	24	21	
BMI (Body mass index)	20.71 ± 2.70	20.50 ± 2.80	0.68
Complications			0.144
Sinus bradycardia	2	0	
Ventricular preexcitation	1	0	
Family history			0.45
Positive	16	16	
Negative	38	32	
Grading of PPH			0.33
Moderate	17	18	
Severe	37	30	

**Table 2:** Locations of hyperhidrosis before surgery.

Hyperhidrosis site	Cases	Percentage (%)
palms	4	3.9
palms and soles	44	42.7
palms and axillae	1	0.9
Palms, axillae and soles	53	53.7
Total	102	100

### Perioperative data

Patients in both groups who received ETS had a 100% efficacy rate. Their hand temperature rise by more than 1°C and their palms were dry on the day of surgery. A thoracic drainage tube was indwelled in 5 patients due to intraoperative pleural adhesion. After surgery, 13 patients including 8 in group A and 5 in group B, developed a slight pneumothorax, resulting in less than 30% unilateral pulmonary compression. In reexamination one month later, gas absorption was observed with chest radiographs despite no special treatment. No serious complications occurred such as severe arrhythmia, Horner syndrome, hemothorax, or cardiac arrest. Between the two groups, there were no significant differences in operative time, intraoperative blood loss, pneumothorax, or any complications (*P* > 0.05) shown in Table 3.

**Table 3:** Perioperative data.

Variables	Group A	Group B	*P* value
Duration of surgery (min)	61.65 ± 19.07	57.19 ± 14.58	0.19
Intraoperative blood loss (ml)	22.09 ± 14.80	24.38 ± 15.73	0.45
Placement of drainage tube			0.556
Yes	3	2	
No	51	46	
Complications			
Pneumothorax	8	5	0.359
Hydrothorax	0	0	
Horner's syndrome	0	0	
Postoperative blood loss	0	0	
Cardiac arrest	0	0	
Efficacy rate	100%	100%	
Time of Hospitalization (day)/ length of hospital stays	3.83 ± 1.87	4.52 ± 2.41	0.11

### Postoperative follow-up

**Figure 2:**
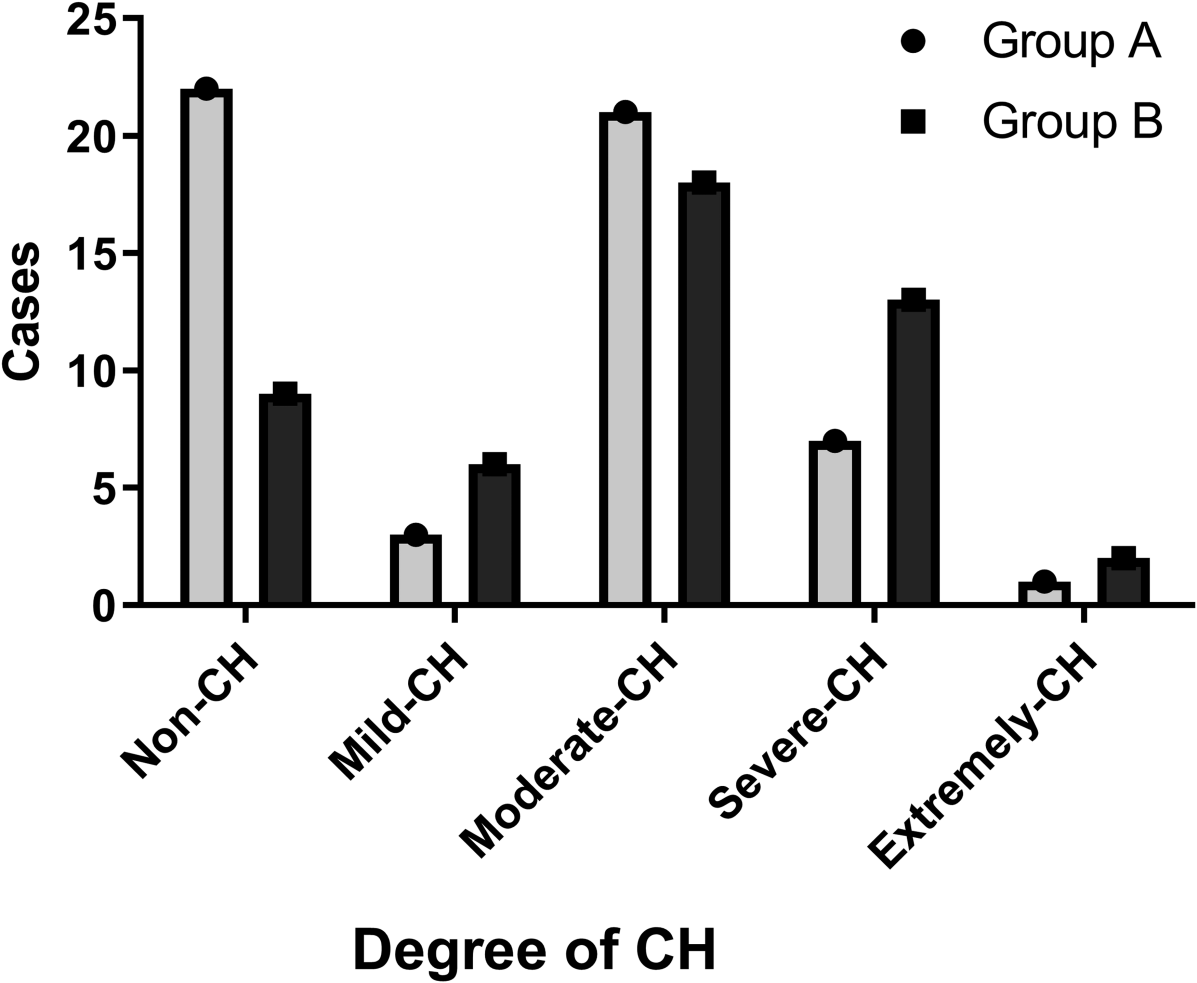
Cases of different degree of CH.

In postoperative follow-up, no statistically significant difference was seen with the surgical satisfaction rate of group A being 98.15% (53/54), and of group B being 95.83% (46/48) (*P* > 0.05). In group A, dry palms were 38.89% (21/54), normal palms were 38.89% (21/54), moist palms were 22.22% (12/54), and no recurrence occurred. In group B, patients with dry palms were 39.58% (19/48), normal 41.47% (19/48), moist 18.75% (9/48), and no recurrence. There was no significant difference in palm status between the two groups (*P* > 0.05). In group A, the CH rate was found to be 59.25% (32/54), mild and moderate CH rate was 44.4% (24/54), severe and extremely severe CH rate was 14.81% (8/54). CH rate in group B was found to be 81.25% (39/48), mild and moderate CH rate was 50% (24/48), severe and extremely severe CH rate was 31.28% (15/48). The incidence of CH was significantly lower in group A than in group B, with a statistically significant difference (*P* < 0.05). The incidence of severe and extremely severe compensatory hyperhidrosis was also significantly lower in group A than in group B, with a statistically significant difference (*P* < 0.05) (Table 4, Figure 2).

**Table 4:** Postoperative follow-up.

Observation indices	Group A	Group B	*P* value
Surgical satisfaction			0.455
No	1	2	
Yes	53	46	
Occurrence of CH			0.019
Yes	32	39	
No	22	9	
Degree of CH			
Mild	3	6	
Moderate	21	18	
Severe	7	13	
Extremely severe	1	2	
Severe + Extremely severe	8	15	0.04
Postoperative palm status			0.905
Dry	21	19	0.552
Normal	21	20	0.457
Moist	12	9	0.427
Recurrence	0	0	

Patients with or without postoperative CH had their palmar status compared. Patients without CH had 22.58% (7/31) dry palms, 61.29% (19/31) normal palms, and 16.13% (5/31) moist palms, compared to patients with CH who had 46.48% (33/71) dry palms, 30.98% (22/71) normal palms, and 22.53% (16/17) moist palms. There was a statistically significant difference in postoperative palmar status between patients with and without CH (*P* < 0.05). According to a subgroup analysis of the three types of postoperative palm status in patients without CH and with CH, the number of patients with dry palm in the CH group was substantially higher than that in the non-CH group, *P* < 0.05 (Table 5).

**Table 5:** Palmar status of patients with or without postoperative CH.

Palmar status	Non-CH group	CH group	*P* value
Dry	7	33	0.014
Normal	19	22	0.018
Moist	5	16	0.004
Recurrence	0	0	0.326

### Postoperative QoL score

The quality of life of 102 follow-up patients was assessed using the QoL score. There were no statistical differences in total QoL score, G1, G2, physiological, social and environmental domains between the two groups (*P* > 0.05), although group A's psychological score was considerably higher than group B's (14.46 ± 2.06 vs. 13.30 ± 1.86). *P* < 0.01) (Table 6).

**Table 6:** Postoperative QoL score.

Variables	Group A	Group B	*P* value
G1	3.78 ± 0.84	3.48 ± 0.85	0.078
G2	3.70 ± 0.84	3.56 ± 0.85	0.400
physiological	13.46 ± 2.17	12.76 ± .1.82	0.084
psychological	14.46 ± 2.06	13.30 ± 1.86	0.004
social	14.59 ± 2.16	14.62 ± 2.30	0.942
environmental	14.86 ± 2.02	14.61 ± 2.24	0.560
Total score of QoL	64.84 ± 8.38	62.34 ± 7.67	0.121

## Discussion

PPH is considered heritable, and its pathogenesis has not been entirely explained. Chen declared the primary pathogenic gene location on chromosome 14q11.2-q13 (9). The quantity of palmar sweat gland attachments in patients with PPH is identical to that in normal people. Therefore, the etiology of PPH is supposed to be connected with the dysfunction in the complex autonomic nervous system and abnormality in emotional center control (10). ETS functions on PPH by disconnecting the related thoracic sympathetic chain to obstruct sympathetic impulses to the palmar sweat glands. Based on our research data of follow-up, the effective rate of ETS treatment was 100% in 102 patients with PPH. Particularly, the symptoms of all patients with palmar hyperhidrosis have improved significantly, whereas a small number of patients with palmar combined axillary or plantar hyperhidrosis had improved in different magnitudes, despite the recurrence of most of the patients in the plantar sweat.

ETS is safe for the treatment of PPH. In this study, all 102 patients were discharged successfully following surgery. Three of them had preoperative arrhythmia; however, no cardiovascular complications were observed after receiving ETS after preoperative pacemaker implanted and radiofrequency ablation. Early postoperative complications of ETS primarily are pneumothorax, hemothorax, intercostal neuralgia, incision infection and others, all of which are temporary and self-limited (11). Pneumothorax is the most frequent complication among them, and there were 13 patients with slight pneumothorax following surgery. Chest x-ray examination revealed that lung compression was less than 30%, and the symptom of chest distress was not obvious. After conservative treatment, pneumothorax was self-absorbed and improved. Though extensive pleural adhesions are contraindications for ETS, preoperative chest CT examination cannot precisely diagnose whether pleural adhesions are present. In this study, 3 patients were found to have extensive pleural adhesions during surgery, and after adding a second incision and indwelling drainage tube, all 3 patients successfully finished the minimally invasive surgery and were discharged from the hospital with successful extubation. Horner syndrome and cardiac arrest are serious complications of ETS. The incidence of Horner syndrome is less than 1%, and some patients heal themselves over time. Cardiac arrest is very rare. Robert and Chow stated the requirement of pacemaker maintenance for intraoperative cardiac arrest and postoperative intractable bradycardia (12, 13). So, changes in heart rate should be closely monitored during and after ETS. None of the 102 patients in the study had serious complications.

Recurrence of postoperative hyperhidrosis and constant sweating of palms directly influence the surgical results. Wet palm and PPH recurrence generally result from unexcised sympathetic nerve activities, which are alternative neurals including Kuntz's nerve, also called rami communicantes (RC) (14–18). RC is primarily accountable for connecting the thoracic sympathetic ganglion and the intercostal nerve. There are four types of variations (19, 20), namely, normal branch (NR), ascending branch (AR), descending branch (DR), and intercostal branch (IR). These variant branches constantly lie during the clipping of the lower sympathetic chain to cause persistent palm-sweating after surgery. On that account, we should ensure not only the complete removal of the sympathetic chain but also maximized excision of the RC. We first fulgurated the trunk and branch of the R4 sympathetic chain. Then simultaneously fulgurated the branch of the R3 sympathetic chain in full measure to counterbalance the potential risk of recurrence caused by simple resection of the lower nerve. Our results exhibited that thoracic R4 sympathicotomy combined with R3 ramicotomy has an identical effect with the simple thoracic R3 sympathicotomy on palmar sweating, both of which had no recurrence. There was an insignificant difference in the proportion of moist palms between the two groups (22.22% vs. 18.75%, *P* = 0.427).

CH is also the most significant factor, with an incidence of 33% -80% influencing patient satisfaction with surgery and postoperative quality of life (21, 22). The mechanism of CH occurrence is still being studied. Some researchers believe that CH occurs because of the interference of the reflex arcs between the sympathetic nervous system and the Hypothalamus, causing impaired sweat control (23). A meta-analysis of sympathectomy in various segments for treating Palmar hyperhidrosis revealed that the incidence of postoperative compensatory hyperhidrosis declined with the decrease in sympathectomy segments (24). This implies that the drier the palm after EST, the higher the possibility of postoperative CH. This study detected a remarkably greater amount of dry palms in all patients with CH than those without CH, which is consistent with previous studies. So how to balance the palm state and the compensatory hyperhidrosis state after EST is a problem that we need to focus on. Kim's study manifested that the high-plane ramicotomy did not escalate the occurrence of CH (25). Lee implemented RC to decrease the occurrence of CH. They compared the R3 ramicotomy with the R3 sympathicotomy in two groups. The R3 ramicotomy had a low incidence of postoperative compensatory hyperhidrosis but a high recurrence rate (26). In our study, the incidence of compensatory hyperhidrosis was 59.33% in the R4 sympathicotomy combined with R3 ramicotomy group which is considerably lower than 81.25% in the R3 sympathicotomy group (*p* < 0.05). There were no considerable differences in postoperative recurrence and palmar status in both groups which indicates that the effectiveness of the modified surgical approach in controlling postoperative CH without influencing surgical results, and this result is consistent with our presumptions and findings in the literature.

QoL score is a scale developed by the World Health Organization to measure the quality of life concerning the modern definition of health (27). Several studies have asserted that the quality of life of patients with PPH after thoracoscopic sympathicotomy improves more remarkably than before (28). The QoL score of patients with PPH was primarily impacted by palmar sweating and psychological and social factors. Though there was no considerable statistical difference in the total score of QoL between the two groups in this study, the psychological score of group A was higher than group B (14.46 ± 2.06 vs. 13.30 ± 1.86, *P* < 0.01). With respect to the psychological distress due to hyperhidrosis, this may be associated with the lower incidence of compensatory hyperhidrosis in group A.

Though the improved surgical approach lowered the incidence of CH, the rate was 59.25%, which was not low, and severe to extremely severe CH still occurred. However, compared to bilateral R3 sympathicotomy, the modified surgical method did not enhance the overall QoL of patients, which was associated with the limitation of QOL score considerably, but the surgical procedure still needs improvement. Besides, this research is a single-centered retrospective study with a limited sample. Remedial effects should be substantiated by multicentric and large-scale prospective studies.

Endoscopic thoracic R4 sympathicotomy combined with R3 ramicotomy was harmless and effective, which successfully decreased the occurrence of CH while ensuring the surgical effectiveness. Meanwhile, low-plane sympathicotomy combined with multi-ramicotomy offers a practicable procedure for PPH treatment.

## Data Availability

The original contributions presented in the study are included in the article/Supplementary Material, further inquiries can be directed to the corresponding author/s.
